# Integrated Genomic and Metabolomic Analysis Illuminates Key Secreted Metabolites Produced by the Novel Endophyte *Bacillus halotolerans* Cal.l.30 Involved in Diverse Biological Control Activities

**DOI:** 10.3390/microorganisms10020399

**Published:** 2022-02-09

**Authors:** Polina C. Tsalgatidou, Eirini-Evangelia Thomloudi, Eirini Baira, Konstantinos Papadimitriou, Aggeliki Skagia, Anastasia Venieraki, Panagiotis Katinakis

**Affiliations:** 1 Laboratory of General and Agricultural Microbiology, Crop Science Department, Agricultural University of Athens, Iera Odos 75, 11855 Athens, Greece; polinatsal@gmail.com (P.C.T.); e.e.thomloudi@gmail.com (E.-E.T.); Angeliki.Skagia@warwick.ac.uk (A.S.); 2 Department of Agriculture, University of the Peloponnese, 24100 Kalamata, Greece; 3 Laboratory of Toxicological Control of Pesticides, Scientific Directorate of Pesticides’ Control and Phytopharmacy, Benaki Phytopathological Institute (BPI), Kifissia, 14561 Athens, Greece; e.baira@bpi.gr; 4 Department of Food Science and Technology, University of the Peloponnese, 24100 Kalamata, Greece; kostas.papadimitriou@gmail.com; 5 Laboratory of Plant Pathology, Crop Science Department, Agricultural University of Athens, Iera Odos 75, 11855 Athens, Greece

**Keywords:** postharvest biocontrol, biosynthetic gene clusters, horizontal gene transfer, plant defense elicitors, grapes, tomato, *Botrytis cinerea*

## Abstract

The endophytic strain Cal.l.30, isolated from the medicinal plant *Calendula officinalis*, was selected among seven *Bacillus* strains with plant growth promoting activity and strong biological potential against the postharvest fungal pathogen *Botrytis cinerea*. Treatment by inoculating Cal.l.30 bacterial cell culture or cell free supernatant on harvested grapes and cherry tomato fruits, significantly reduced gray mold disease severity index and disease incidence. Based on 16S rRNA sequence analysis and whole genome phylogeny, Cal.l.30 was identified as *Bacillus halotolerans*. Genome mining revealed that *B. halotolerans* Cal.l.30 is endowed with a diverse arsenal of secondary metabolite biosynthetic gene clusters (SM-BGCs) responsible for metabolite production with antimicrobial properties. A sub-set of the identified SM-BGCs (mojavensin A, ‘bacillunoic acid’) appears to be the result of recent horizontal gene transfer events. Its genome was also mined for CAZymes associated with antifungal activity. Further UHPLC-HRMS analysis indicated that Cal.l.30 synthesizes and secretes secondary metabolites with antimicrobial activity, including the lipopeptides, fengycin, surfactin and mojavensin A, bacillaene isoforms, L-dihydroanticapsin and bacillibactin. Other compounds with known antimicrobial activity were also detected, such as azelaic acid, 15- hydroxypentadecanoid acid and 2-hydroxyphenylacetic acid. The genomic and metabolomic features of the *B. halotolerans* Cal.l.30 provided new perspectives on the exploitation of novel *Bacillus* sp. as a biocontrol agent.

## 1. Introduction

The extensive use of chemical pesticides, herbicides and fertilizers has a significant impact on environmental pollution. Due to this, there has been much attention given to the study and implementation of sustainable, efficient and environmentally friendly products in agriculture [1]. The application of beneficial microorganisms, and especially bacteria, has gained a lot of interest due to their multiple plant growth promoting and plant protection mechanisms, making it necessary to find strong and effective biological control agents (BCAs) [2]. Plant growth promoting rhizobacteria (PGPR) and plant growth promoting endophytic bacteria (PGPEB) interact directly with their host plant and compete with other microbial antagonists, either in the rhizosphere or endophytically, and are considered as useful tools to develop efficient biocontrol strategies [1,3].

Plant associated bacteria of the genus *Bacillus* have been extensively studied for their successful antagonistic activity against a wide range of plant pathogenic microorganisms, including bacteria, fungi and viruses [4,5]. *Bacillus* species are known for their potential to biosynthesize and secrete multiple secondary metabolites, being the fundamental producers of lipopeptide compounds [6,7]. Depending on the biosynthetic pathway, the bioactive compounds are separated either into small molecular peptides synthesized ribosomally (ribosomal peptides, RPs), into peptide compounds synthesized by the non-ribosomal pathway (non-ribosomal peptides, NRPs), such as lipopeptides (LPs) and polyketides (PKs), or into hybrids of PKs and NRPs, such as bacillaene [8]. Genes involved in the biosynthesis of antibiotics and secondary metabolites are organized in clusters and encode multifunctional enzyme complexes, with the gene clusters polyketide synthase (PKS) and non-ribosomal peptide synthetase (NRPS) being the most well studied [9]. The bioactive secondary metabolites of *Bacillus* species biosynthesized by the PKS/NRPS pathways include surfactin, iturin, fengycin, mycosubtilin, bacilysin and bacillaene compounds [8].

The most studied and exploited *Bacillus* species are *Bacillus subtilis, Bacillus velezensis* and *Bacillus amyloliquefaciens*, due to their suppressive effect against several plant diseases [5,10]. However, an interesting but less studied *Bacillus* species that has recently gained interest for its plant growth effect and antimicrobial activity is *Bacillus halotolerans* [11,12,13]. As other *Bacillus* species, many *B. halotolerans* strains can biosynthesize and secrete several bioactive secondary metabolites and lytic enzymes, suppressing multiple phytopathogenic fungi such as *Botrytis cinerea, Rhizoctonia bataticola, Fusarium oxysporum, Alternaria alternate, Sclerotinia sclerotiorum* and *Phytophthora infestans* under in vitro or ex vivo conditions [11,12,14,15,16,17,18].

The phytopathogen *B. cinerea* is a potent necrotrophic fungus able to infect a significant number of fruits and berries, such as strawberry, tomato, grape, kiwi, cucumber, apples, peaches, etc., causing the postharvest gray mold disease [19]. The application of beneficial bacterial strains on plant tissues is an important method of selecting potential BCAs able to suppress phytopathogens causing postharvest diseases. The ex vivo biological control method places both the BCA candidate and the phytopathogenic microorganisms in more natural environmental conditions, being an alternative approach to study the mechanisms for combating postharvest diseases in fruits and vegetables [10,20]. The detached fruit method has been implemented either as an initial selection method, or after the dual culture method for the detection of competitive BCAs with strong biocontrol ability in vitro [21,22,23,24]. 

The present study focuses on *B. halotolerans* strain Cal.l.30, a strong BCA candidate among four *B. halotolerans* strains (Cal.f.4, Cal.l.11, Cal.f.2.1, Cal.r.11), one *B. velezensis* (Cal.r.29) and one *B. subtilis* strain (Cal.r.19), as previously isolated from different plant parts of the medicinal plant *C. officinalis* [25]. We therefore studied its antagonistic activity for the suppression of the postharvest pathogen *Botrytis cinerea*, under in vitro and ex vivo conditions on detached tomato fruit and grape berries. Furthermore, genome and metabolomic analyses revealed the presence of different biosynthetic gene clusters (BGCs) of several secondary metabolites and the potential of strain Cal.l.30 to biosynthesize and secrete them. 

## 2. Materials and Methods

### 2.1. Dual Culture Assay 

For the detection of antagonistic activity of the seven selected endophytic bacterial strains against the phytopathogenic fungus *B. cinerea*, an in vitro dual culture method was performed as described by Nifakos et al., 2021 [26]. A mycelial disc of 5-mm diameter from the edge of a 7-day-old colony of the phytopathogenic fungus was cut out using a sterile cork borer and placed onto an NA plate. After 18 h of bacterial incubation in Nutrient Broth (NB), 10 µL of bacterial culture was inoculated at a 3 cm distance from the fungus. NA plates inoculated with the antagonistic bacterial strains and the phytopathogen as well as the phytopathogen alone (control plate), were maintained at 25 °C for 10 d. The antagonistic effect was detected by measuring the radial growth of *B. cinerea* in control plates and after interaction with the selected bacterial strains. The inhibition rate of mycelium growth was calculated using the formula

(1) $$\%\;\mathrm{Inhibition}\;\mathrm{rate}\;\mathrm{of}\;\mathrm{fungus}=\frac{\left(A1-A2\right)}{A1}\times 100$$

where A1 represents the colony diameter of control and A2 represents the colony diameter of treatment [27,28].

### 2.2. Drop Collapse Assay

Drop collapse assay for the seven selected bacterial strains was performed with small modifications, as described by Townsley et al. (2018) [29], in order to determine their ability to biosynthesize and secrete biosurfactants in liquid culture. Briefly, bacterial cultures after grown for 2 days at 28 °C in Nutrient Broth were centrifuged at 10,000 rpm for 10 min. A 25 μL aliquot of the supernatant was placed on the parafilm, and after 5 min the droplet was examined visually. A small quantity (1 μL) of Evans Blue dye was added to each droplet to better visualize and capture the presence of biosurfactants affecting the droplet shape. Distilled water was used as negative control. 

### 2.3. Detached Fruit Assay 

The selected endophytic bacterial strain Cal.l.30 was further evaluated for its antagonistic activity against *B. cinerea* on tomato fruit (*Solanum lycopersicum* L. cv. Lobello) and grape berries (*Vitis vinifera* L. cv. Sultanina), using the ex vivo modified detached fruit assay as described by Shi and Sun, 2017 [30]. Tomato fruits and grape berries were surface sterilized with 3% *v/v* NaClO for 15 min, rinsed four times with sterilized dH_2_O, air dried for 1 h at room temperature and wounded with a sterile cork borer (3 mm diameter) in one side. An aliquot of 10 μL of bacterial culture (1 × 10^8^ CFU/mL), 10 μL of bacterial cell free supernatant of the same culture and 10 μL of sterilized distilled water as control treatment were inoculated on the surface of each wound and incubated at room temperature for 1 h before applying the fungus. Conidial suspension was prepared by flooding Potato Dextrose Agar (PDA) plates of a 10-day-old solid culture with sterilized dH_2_O to gently remove the conidia and adjust the concentration approximately to 1 × 10^5^ spores/ml. Finally, 10 μL of fungal spore suspension was injected into each wound. Inoculated tomato fruit and grape berries were placed into plastic boxes to maintain high relative humidity (approximately 80%) and incubated in a dark growth chamber at 25 °C for 5 d. The experiment was conducted three times (20 fruit/ replicate). The percentage of infected tomato fruits and grape berries by *B. cinerea* were calculated to assess Disease Incidence (DI) according to the following equation

(2) $$\%\;\mathrm{Disease}\;\mathrm{Incidence},\;\left(\mathrm{DI}\right)=\frac{\mathrm{Number}\;\mathrm{of}\;\mathrm{infected}\;\mathrm{fruits}}{\mathrm{Total}\;\mathrm{number}\;\mathrm{of}\;\mathrm{fruits}}\times 100$$

Growth area of the phytopathogenic fungus on each fruit surface was calculated using ImageJ software analysis tool in order to determine Disease Severity (DS) using the following equation

(3) $$\%\;\mathrm{Disease}\;\mathrm{Severity},\;\left(\mathrm{DS}\right)=\frac{\mathrm{Infected}\;\mathrm{fruit}\;\mathrm{area}}{\mathrm{Total}\;\mathrm{fruit}\;\mathrm{area}}\times 100$$

From the calculated disease severity, a percentage scale of the disease severity (Rating scale) is obtained, based on the modified Horsfall–Barratt scale method [31,32,33]. Disease Severity Index (DSI) was scored on a 0 to 9 rating scale, with 0 = healthy fruits, 1 = 1–10%, 3 = 11–25%, 5 = 26–50%, 7 = 51–75% and 9 = >75% infected fruit area and was calculated according to the formula

(4) $$\%\;\mathrm{Disease}\;\mathrm{Severity}\;\mathrm{Index},\;\left(\mathrm{DSI}\right)=\frac{\sum \left(n\times i\right)}{N\times Z}\times 100$$

where n: number of fruit in a specific value of disease rating scale, i: the corresponding value of the scale, N: total number of fruit, Z: highest value of disease rating scale. 

### 2.4. Colonization on Wounded Grape Berries and Tomato Fruits

To evaluate the colonization of bacterial strain Cal.l.30 when inoculated alone on the wounds of grape berries and tomato fruits, tissue samples were cut out of the surfaces with a sterile cork borer (0.4 cm diameter and 0.4 cm deep) and transferred into a falcon tube containing 10 mL of sterile phosphate-buffered saline (PBS: KH_2_PO4, 0.27 g/L; KCl 0.2 g/L; NaCl 8 g/L; Na_2_HPO_4_ 1.42 g/L; pH 7.0). After shaking for 1 h at 200 rpm, the suspensions were diluted by 10- fold serial dilutions, and 100 μL of the last dilution was plated in NA supplemented with 50 μg/mL cycloheximide and was then incubated at 30 °C for 48 h in order to calculate bacterial colony-forming units. Four samples were collected of each fruit at 0, 24, 48, 72, 96 and 120 h post inoculation and the experiment was repeated independently three times.

### 2.5. Extraction of Agar-Diffusible Secondary Metabolites of Cal.l.30

The protocol used for the extraction of the agar-diffusible secondary metabolites (ADSM) of Cal.l.30 either secreted when grown singly (ADSM1), or during interaction with *B. cinerea* (ADSM2) was conducted as previously described by Nifakos et al., 2021 [28]. Firstly, an aliquot of 200 μL of pre-grown bacterial culture of strain Cal.l.30 was inoculated in NA plates (1.5% (*w/v*) agar) in an artificial well (0.5 cm × 6 cm) against a mycelial disc (5 mm diameter) of the 10-day-old phytopathogenic fungus *B. cinerea*. Plates inoculated only with the bacterial strain were used as control treatment. All NA plates were maintained in 25 °C for 6 d until the creation of a clear inhibition zone between the fungus and strain Cal.l.30. Afterwards, NA medium blocks from the intermediate zone of inhibition created during interaction, as well as the zone in front of the bacterial control treatment, were removed and cut into smaller pieces. Ethyl acetate and 0.1% formic acid were added to NA pieces and thoroughly mixed. Samples were transferred to a water-bath sonicator (Elmasonic S30H, Elma Schmidbauer GmbH, Germany) and were incubated for 30 min at room temperature. After separation of the organic phase, samples were transferred to a speed vacuum evaporator (Rotavapor R-114, BÜCHI Labortechnik AG, Flawil, Switzerland) to dry. Finally, samples were dissolved in HPLC grade methanol, filtered and stored at −80 °C, until further use.

### 2.6. Antagonistic Activity of Agar-Diffusible Secondary Metabolites of Cal.l.30 against B. cinerea In Vitro

Antagonistic activity of agar-diffusible secondary metabolites of Cal.l.30 (ADSM1 and ADSM2) against *B. cinerea* mycelia growth was evaluated by the disc diffusion method. A mycelia disc (5-mm diameter) from the colony edge of a 10-day-old *B. cinerea* culture was applied in the center of a NA plate and three round filter papers were placed approximately at 1 cm distance around the phytopathogenic fungus. An aliquot of 20 μL of each ADSM1 and ADSM2 was inoculated in the two filters while in the third filter 20 μL of methanol was applied as negative control. Plates were incubated at 25 °C for 5 d and antimicrobial activity was determined by the creation of an inhibition zone between the filters and *B. cinerea*. 

### 2.7. TLC-bioautography Assay of Cal.l.30 Secreted Compounds 

Thin-layer chromatography (TLC) in combination with bioautography assay of the agar-diffusible secreted compounds of Cal.l.30 was conducted as previously described by Nifakos et al., 2021 and Calvo et al., 2019 [26,34]. ADSM1 and ADSM2 samples were separated by TLC after spotted in an aliquot of 20 μL onto silica gel 60 F254 TLC aluminum sheets (20 × 20 cm; layer thickness, 0.20 mm; Merck, Darmstadt, Germany) with mobile phase consisting of chloroform–methanol–water at 65:25:4, *v/v/v*. After drying the TLC plate at room temperature, the bioautography assay was performed with *B. cinerea* being the indicator strain. PDA (0.8% agar *w/v*) semi-solid medium inoculated with approximately 10^5^ spores/ mL of *B. cinerea* was poured onto the TLC plate and then incubated at 25 °C overnight. The inhibition zones indicating the presence of antifungal compounds became visible after spraying with MTT (3-(4,5-dimethylthiazol-2-yl)-2,5-diphenyltetrazolium bromide) at a concentration of 5 mg/ml. The R*f* values were calculated using the formula

(5) $$Rf\;\mathrm{value}=\frac{\mathrm{Distance}\;\mathrm{travelled}\;\mathrm{by}\;\mathrm{the}\;\mathrm{solute}}{\mathrm{Distance}\;\mathrm{travelling}\;\mathrm{by}\;\mathrm{the}\;\mathrm{solvet}}$$

### 2.8. Ultra-High Performance Liquid Chromatography Orbitrap High-Resolution Mass Spectrometry Analysis

The chemical profiling of ADSM1 extract was performed as previously described by Nifakos et al., 2021 [28]. Specifically, the Q Exactive Orbitrap platform (Thermo Fisher Scientific, San Jose, CA, USA) was coupled to a Dionex Ultimate 3000 UHPLC system (Thermo Scientific™ Dionex™, Sunnyvale, CA, USA) and the separation was performed using a Hypersil Gold UPLC C18 (2.1 × 150 mm, 1.9μm) reversed phased column (Thermo Fisher Scientific, San Jose, CA, USA). For mass detection, two electrospray ionization modes were carried out, the positive (ESI+) and negative (ESI−). Eluent A consisting of ultrapure water with 0.1% formic acid and eluent B consisting of acetonitrile were used in a gradient mode of 30 min, under the following program: 0–21 min: 95% A–5% B; 21–24 min: 5% A–95% B; 24–30 min: 95% A–5% B, at 0.22 mL/min flow rate. Data acquisition was performed on a mass range of 115–1500 Da on profile mode. The HRMS parameters for both ion modes were set as follows: spray voltage, 2.7 kV; capillary temperature, 350 °C; aux. gas heater temperature, 50 °C; sheath gas flow, 40 arb. units; S-lense R*f* level, 50 V; aux gas flow, 5 arb. units. The resolution for full scan analysis was set on 70,000, whereas for the data dependent acquisition mode the resolution was 35,000 allowing for MS/MS fragmentation of the three most intense ions. Stepped normalized collision energy was set at 35, 60 and 100. The column temperature was set at 40 °C and the sample tray temperature at 4 °C.

The resulting data were processed through the Compound Discovered version 2.1 (Thermo Fisher Scientific, San Jose, CA, USA). The online mzCloud library, the public chemical database PubChem (NCBI) and the Norine platform were used for metabolite annotation, taking into consideration the isotopic pattern and applying *m/z* tolerance of 5 ppm.

### 2.9. Genome Sequencing

Genome sequencing of *B. halotolerans* strains Cal.l.30, Cal.f.4 and Cal.l.11 was conducted as previously described by Nifakos et al., 2021 [26]. Briefly, genomic DNA of the bacterial strains was isolated from an overnight culture using PureLink® Genomic DNA Mini Kit (Thermo Fisher Scientific, Carlsbad, CA, USA) and sequenced by SNPsaurus (Eugene, OR, USA) using an Illumina HiSeq 2000 platform, generating 2 × 150-bp paired-end reads. A library was created using the Nextera XT DNA Library Prep Kit. Trimming of adaptors was performed with BBDuk and assembly with SPAdes-3.12.0 using default parameters [35]. This workflow generated a total of 1.546.685 (Cal.l.30), 1.251.500 (Cal.f.4), 1.442.900 (Cal.l.11) trimmed paired reads with coverage >60-fold. The final de novo genome of strain Cal.l.30 (NCBI accession: JAEACK000000000) assembled in 9 scaffolds with size of 4.206.058 bp, of strain Cal.f.4 (NCBI accession: JAEACI000000000) assembled in 12 scaffolds with size of 4.283.396 bp, Cal.l.11 (NCBI accession: JAEACJ000000000) assembled in 11 scaffolds with size of 4,212,585 bp.

The whole genome data of strains Cal.l.30, Cal.f.4 and Cal.l.11 have been deposited at DDBJ/EMBL/GeneBank under the accession numbers JAEACK000000000, JAEACI000000000 and JAEACJ000000000, respectively. The identification of all three strains was established using the Type (Strain) Genome server (TYGS) platform (https://tygs.dsmz.de, accessed on 15 December 2021) [36] and by calculating the values of digital DNA-DNA hybridization (dDDH) using the genome to genome distance calculator (GGDC 2.1) [37] and the values of orthologous average nucleotide identity (OrthoANI) [38].

### 2.10. Functional Genome Analysis

The secondary metabolite BGCs were identified using the online server antiSMASH 6.0 [39]. The ClusterBlast and KnownClusterBlast modules integrated into antiSMASH 6.0 were also used for comparative gene cluster analysis based on the NCBI GenBank and the ‘Minimum Information about a Biosynthetic Gene Cluster’ (MIBiG) data standard, respectively [39,40,41]. The server dbCAN2 and the combination of HMMER (E-Value < 1 × 10^−15^ coverage > 0.35), DIAMOND (E-Value < 1 × 10^−102^) and Hotpep 117 (Frequency > 2.6, Hits > 6 tools, were used to predict the carbohydrate-active enzymes (CAZymes) in the core genome [42]. CAZyme domains predicted by at least two of the three algorithms (DIAMOND, HMMER and Hotpep) employed by dbCAN2 were considered as a true annotation for carbohydrate-active enzymes [42].

### 2.11. Statistical Analysis

For statistical analysis, IBM SPSS Statistics for Windows, version 25 (IBM Corp., Armonk, NY, USA) software was used while plots were carried out with Sigma Plot, version 12.0 (Systat Software, San Jose, CA, USA). The heatmaps were created using Microsoft Excel 2010. To evaluate bacterial population, data were transformed to the logarithmic scale prior to Tukey analysis (*p*-value < 0.05). Data expressed as percentages were arcsine transformed prior to Tukey analysis (*p*-value < 0.05).

## 3. Results

### 3.1. Biological Control Activity of Bacillus Strains from Calendula officinalis

**Figure 1:**
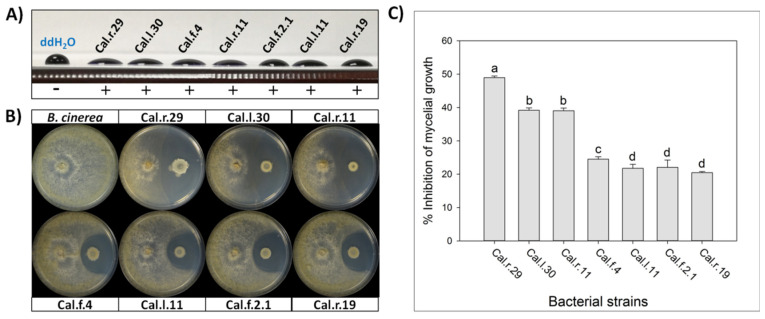
(**A**) Detection of secreted biosurfactants in the cell free supernatant of the seven studied bacterial strains using drop collapse assay. (**B**) Direct antifungal activity of endophytic *Bacillus* strains against *B. cinerea* by an in vitro dual culture assay. (**C**) Percentage of inhibition of mycelial growth (IMG) after each bacterial formulation against *B. cinerea* in vitro. Data values represent the mean of 4 biological replicates ± SD. Letters indicate the statistical difference between treatments after Tukey analysis (*p* < 0.05).

All seven selected *Bacillus* isolates were tested for biosurfactants secretion. The cell free supernatant of all studied strains showed excellent drop collapse ability with the drop flattening out to different extend among the different strains, indicating adequate biosurfactant production (Figure 1).

The selected endophytic bacterial strains of the genus *Bacillus* (Cal.r.29, Cal.l.30, Cal.l.11, Cal.f.4, Cal.r.11, Cal.f.2.1 and Cal.r.19), presented a strong antagonistic activity against *B. cinerea* in a dual culture assay, until after 10 d of co-inoculation with the fungus (Figure 1B). The antagonistic strains suppressed *B. cinerea* mycelial growth generating a clear inhibition zone of differential width, by an in vitro dual culture assay, indicating adequate production of agar-diffusible antifungal metabolites by all studied bacterial strains (Figure 1B). The radial mycelial growth of *B. cinerea* towards the antagonistic isolates showed significant differences between them, with the highest inhibition rate observed for strain Cal.r.29 (48.95%). Strains Cal.l.30 and Cal.r.11 followed with the second highest inhibition rates at 39.18% and 39.02%, respectively, while strain Cal.f.4 presented the third highest value (24.49%) (Figure 1C). Finally, strains Cal.l.11, Cal.f.2.1 and Cal.r.19, presented the lowest inhibition rates in comparison to the others with values at 22.05%, 21.75% and 20.45%, respectively (Figure 1C). Similar results were also observed when inoculated against phytopathogenic fungi *Rhizoctonia solani* and *Fusarium oxysporum* f.sp. *lycopersici* under in vitro dual culture assays.

### 3.2. Biological Control Potential of Endophytic Bacillus Strain Cal.l.30 against Botrytis cinerea

**Figure 2:**
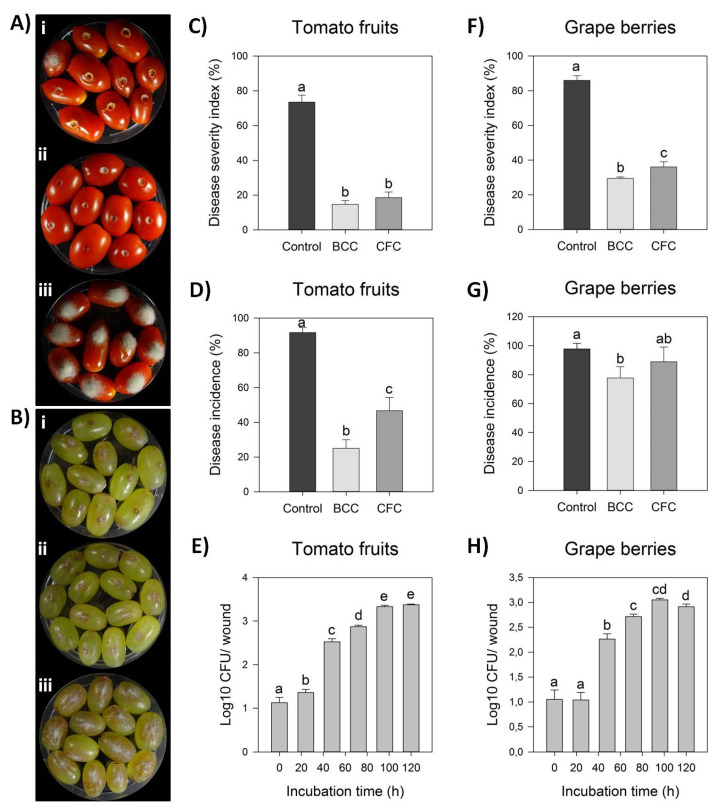
Antagonistic activity of endophytic bacterial strain Cal.l.30 against gray mold disease on tomato fruits and grape berries. (**A**,**B**) Suppression of gray mold disease after ex vivo formulation on grape berries and tomato fruits, respectively, after liquid bacterial cell culture (BCC) (**i**), liquid cell free culture (CFC) (**ii**) and individual formulation of *B. cinerea* as control treatment (**iii**). (**C**,**D**) Disease severity index (%) and Disease Incidence (%) on tomato fruits, respectively. (**E**,**F**) Disease severity index (%) and Disease incidence (%) on grape berries, respectively. Bars represent the mean ± SD of 3 independent biological replicates. Letters indicate the statistical differences between treatments at 4 days after inoculation (DAI) after Tukey analysis (*p* < 0.05). (**G**,**H**) Time growth curve of Cal.l.30 (Log10 CFU/ wound) after inoculation in tomato fruit and grape berry wounds (one wound/ tomato or grape), respectively, without the presence of phytopathogenic fungus *B. cinerea*. Bars represent the mean ± SD of 4 independent biological replicates after Tukey analysis. Letters indicate the statistical difference per hour (*p* < 0.05).

Strain Cal.l.30 was selected among others of the dominant endophytic *B. halotolerans* species for further study on its antagonistic activity against *B. cinerea*. Bacterial strain Cal.l.30 presented a strong inhibitory effect against the postharvest pathogenic fungi, as observed after ex vivo inoculation on grape berries and tomato fruits (Figure 2A,B). Strain Cal.l.30 was able to successfully colonize both of the fruits tested, presenting a high density until the fifth day of observation (Figure 2E,H). 

The tomato fruits when inoculated for five days only with the presence of *B. cinerea* as negative control, presented fruits with extremely severe mycelial growth on top of them, while grape berries developed brown lesion and visible mycelia growth around the point of formulation (Figure 2(Aiii,Biii)). Strain Cal.l.30 suppressed fungal growth after inoculation of liquid bacterial cell culture (BCC) as well as liquid cell free culture (CFC) on both grape berries and tomato fruits (Figure 2(Ai,ii,Bi,ii)). 

However, treatment with BCC (liquid bacterial cell culture) containing both living bacterial cells and multiple secreted secondary metabolites/ biosurfactants was more efficient than CFC in suppressing gray mold in both fruits studied. Specifically, BCC treatment on tomato fruit significantly reduced disease incidence by 14.63% in comparison to CFC inoculation (18.52%) and the negative control (73.52%), whereas disease severity index was equally reduced after both BCC and CFC formulations (Figure 2A,B). As described in Figure 2G, infected grape berries were significantly reduced in both treatments, with disease incidence being 77.78% and 88.89% after BCC and CFC inoculation, respectively, in comparison to the control treatment (97.78%). In addition, disease severity index was also significantly reduced in both BCC and CFC treatments by 28.89% and 33.33%, respectively, in comparison to the control treatment (85.93%) (Figure 2F).

### 3.3. Genomic Features and Taxonomic Classification of Cal.l.30, Cal.f.4 and Cal.l.11

**Figure 3:**
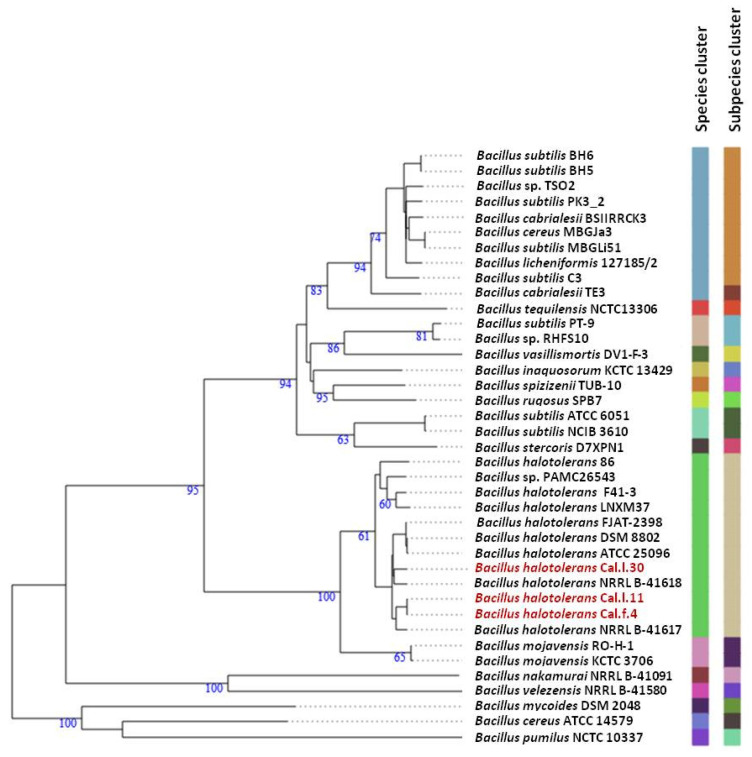
Whole genome phylogenomic tree of *Bacillus* strains closely related to *B. halotolerans* strains Cal.l.30, Cal.f.4 and Cal.l.11, constructed using the type (strain) genome server—TYGS. The numbers at the branches are pseudo-bootstrap support values inferred from 100 replicates. The two colored columns to the right of each name refer to the genome-based species and subspecies clusters, respectively, as determined by dDDH cut-off of 70 and 79%, respectively. *B. halotolerans* strains Cal.l.30, Cal.l.11 and Cal.f.4 are highlighted in red.

**Figure 4:**
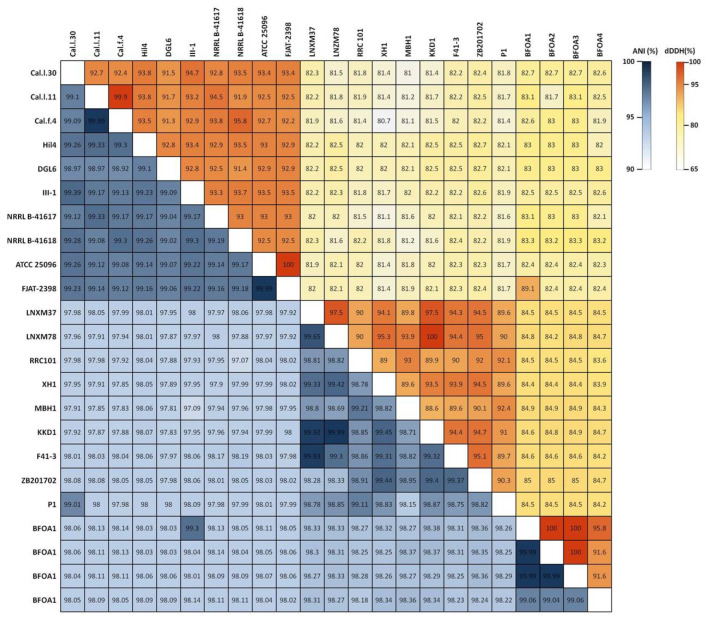
Heatmap of Average Nucleotide Identity (ANI) and digital DNA–DNA Hybridization (dDDH) scores among *B. halotolerans* strains.

To ascertain the taxonomic position of bacterial strains Cal.l.30, Cal.f.4 and Cal.l.11, phylogenetic analysis using whole genome sequences was conducted using the Type (strain) Genome Server (TYGS) bioinformatics platform (https://tygs.dsmz.de, accessed on 15 December 2021). The data indicated that strains Cal.l.30, Cal.f.4 and Cal.l.11 are closely affiliated to *B. halotolerans* species (Figure 3). To confirm this classification, all three genomes were further compared by alignment-based ANI and digital DDH to other genomes of *B. halotolerans* strains. The dDDH values between strain Cal.l.30, Cal.f.4 and Cal.l.11 and other *B. halotolerans* strains ranged between 80.70% and 94.70%, indicating that all three strains are closely affiliated to *B. halotolerans* species as shown in Figure 4. The range between 97.91 and 99.33% of ANI values showed agreement between the two methods for species discrimination and established classification of strains Cal.l.30, Cal.f.4 and Cal.l.11 as *B. halotolerans*. Although, the *B. halotolerans* Cal.l.30 isolate demonstrated a very high level of genome conservation with *B. halotolerans* Cal.l.11 and Cal.f.4, with ANI and dDDH values exceeding 99% and 92%, respectively, a close inspection/comparison of the annotated genes revealed that the genes encoding the proteins of type I restriction-modification DNA-methyltransferases hsdMSR and Mrr proteins (cryptic type IV restriction endonucleases with specificity for methylated DNA), were exclusively found in Cal.l.30.

### 3.4. Genomic Insights into the Antifungal Activity and Biocontrol Potential of Cal.l.30

To gain an insight of the secondary metabolite biosynthetic gene clusters (SM-BGCs) associated with the biosynthesis of metabolites with antimicrobial properties of *B. halotolerans* strain Cal.l.30, a bioinformatic approach based on the antiSMASH and ClusterBlast algorithm platform was used.

**Figure 5:**
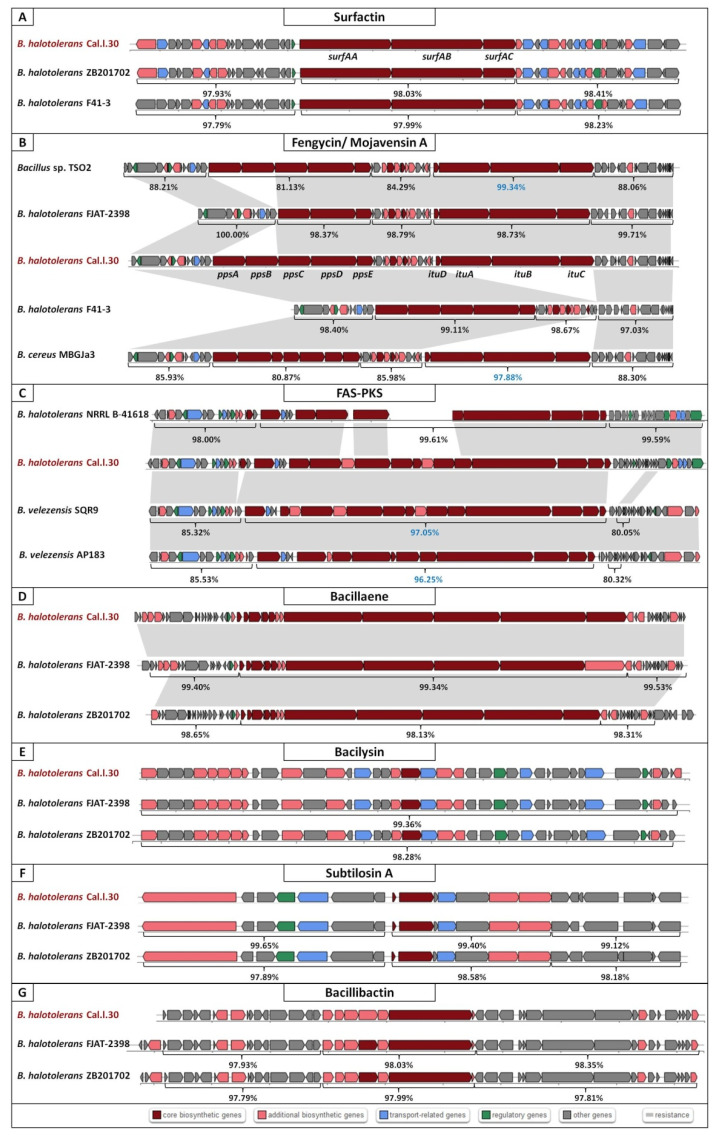
Secondary metabolite biosynthetic gene clusters (BGCs) of *Bacillus halotolerans* Cal.l.30 using antiSMASH. Eight BGCs for the possible production of secondary metabolites: (**A**) Surfactin, (**B**) Mojavensin A/ Fengycin, (**C**) FAS-PKS, (**D**) Bacillaene, (**E**) Bacilysin, (**F**) Subtilosin A and (**G**) Bacillibactin were predicted. Genes with different color represent different functions as described in the colored blocks.

AntiSMASH genome mining for SM-BGCs revealed that *B. halotolerans* Cal.l.30 harbors 10 BGCs, indicating that a large proportion (12.56%) of its genome is dedicated for the biosynthesis of secondary metabolites (Table 1 and Figure 5A–G). Among the predicted BGCs, four NRPS clusters, associated with the synthesis of surfactin, fengycin, bacillaene and bacillibactin, one RiPP cluster, one subtilosin A and one bacilysin gene cluster, were detected (Table 1). BGC of mojavensin A was not present as an individual cluster but was detected adjacent to the fengycin gene cluster. The remaining BGCs, including this of kalimantacin A (bearing only 20% resemblance to the BGC encoding kalimantacin A), appear to be involved in the biosynthesis of metabolites of unknown function.

Genome mining using antiSMASH with the aid of ClusterBlast algorithm showed that the kalimantacin A BGC has a high similarity to a novel FAS-PKS (Fatty Acid Synthases-Polyketide Synthases) biosynthetic gene cluster identified in *B. velezensis* SQR9 and, therefore, will be referred to as FAS-PKS BGC (Figure 5C). Furthermore, a large part of the core genes of the Cal.l.30 FAS-PKS BGC was identified in a BGC of *B. halotolerans* NRLLB-41618 (Figure 5C). Using the Cal.l.30 BGC nucleotide sequences as a Blastn query in the NCBI database, it was realized that the core genes of Cal.l.30 in comparison to strains’ SQR9, AP183 and NRLLB-41618 novel BGCs were collinearly homologous with a high similarity of 97.05%, 96.25% and 99.61%, respectively. In contrast, the up and downstream flanking DNA regions of Cal.l.30 FAS-PKS core BGC in comparison to those of the SQR9 strain, were partly identified and showed a relatively less nucleotide identity, ranging from 85.32% to 85.53% (upstream) and 80.05% to 80.32% (downstream), respectively (Figure 5C). However, in strain NRLLB-41618 both down and upstream flanking DNA regions were highly homologous with the corresponding genes of Cal.l.30 with a range of 98.00% and 99.59, respectively (Figure 5C). 

Furthermore, analysis of Cal.l.30 fengycin’s gene cluster revealed that adjacent to the fengycin/plipastatin BGC, there is a BGC encoding for the biosynthesis of the iturinic type lipopeptide, known as mojavensin A (Figure 5B). Genome mining (antiSMASH analysis) revealed that the recently published genomes of *B. halotolerans* strains F41-3 [43], ZB201702 [44] and KKD1 [44] harbor only the plipastatin BGC, while *B. halotolerans* FJAT-2398 harbors part of the core genes (ppsC, ppsD and ppsE) involved in the biosynthesis of fengycin/plipastatin but also the whole BGC for the biosynthesis of mojavensin A (Figure 5B). The up and downstream flanking DNA regions of Cal.l.30 and FJAT-2398 mojavensin A’s core BGC are found upstream of the F43-1 plipastatin core BGC joined in a sequential mode (Figure 5B). In addition, *Bacillus* sp. TSO2 *and Bacillus cereus* MBGJa3 carry both plipastatin/fengycin and mojavensin A BGCs. The mojavensin A biosynthetic gene cluster in strain Cal.l.30 is homologous to TSO2 and MBGJa3 corresponding BGCs, with similarity rates of 99.34% and 97.8%, respectively, (Figure 5B). Phylogenetic analysis indicated that both *Bacillus sp*. TSO2 and *B. cereus* MBGJa3 are classified as *Bacillus cabrialesii*, a bacterial species phylogenetically diverse from *B. halotolerans* (Figure 3). 

Further genome mining revealed that strain Cal.l.30 harbors the genes alsS, alsD, ilvB, ilvH and bdhA involved in the biosynthesis of the well-known metabolites, 3-hydroxy-2-butanone and 2, 3-butanediol that act as elicitors and promote antimicrobial activity by inducing systemic resistance against plant pathogens [45].

### 3.5. Predicted Secondary Metabolite BGC Richness in B. halotolerans Genomes

**Figure 6:**
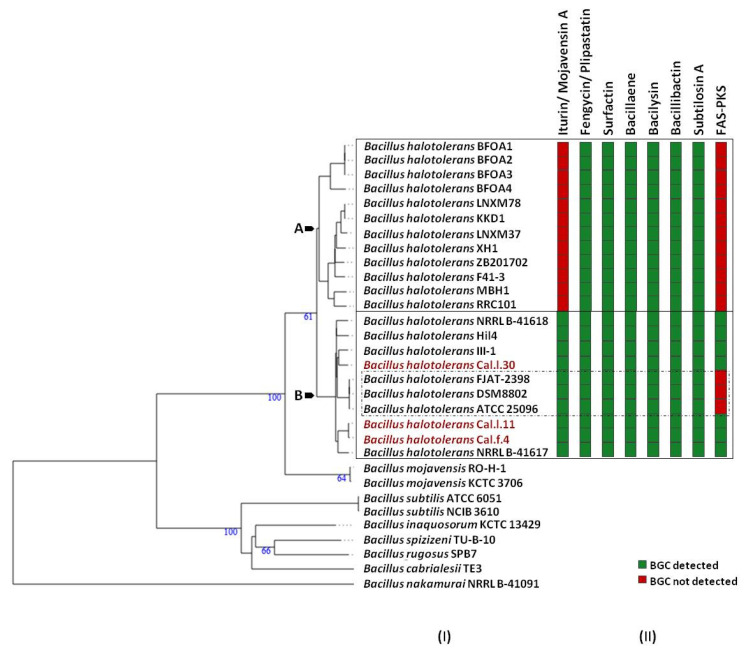
The secondary metabolic gene clusters evolution. (**I**) Whole genome phylogeny of *B. halotolerans* strains highlighting the endophytic bacterial strains Cal.l.30, Cal.f.4 and Cal.l.11. The numbers above branches are pseudo-bootstrap support values from 100 replications. Inferred clusters are for *B. halotolerans* strains harboring (Cluster B) or lacking (Cluster A) the iturin/ mojavensin A BGC. (**II**) Number and distribution heatmap of the secondary metabolite biosynthetic gene clusters (iturin/ mojavensin A, fengycin/ plipastatin, surfactin, bacillaene, bacilysin, bacillibactin, subtilosin A and FAS-PKS) detected (green) or not (red) among *B. halotolerans* strains. *B. halotolerans* strains Cal.l.30, Cal.l.11 and Cal.f.4 are highlighted in red.

To determine which secondary metabolites are encoded in the *B. halotolerans* strains genome, we used antiSMASH 6.0 and ClusterBlast analysis to identify the BGCs in 21 sequenced genomes (Figure 6). Because in some strains BGC is spread in two or more contigs and estimating the precise cluster boundaries is a critical step when computationally comparing BGCs, all these clusters detected by antiSMASH were manually assembled using the most similar cluster identified in antiSMASH ClusterBlast analysis [46,47]. AntiSMASH outputs indicated that all the *B. halotolerans* strains harbor fengycin, surfactin, bacillaene, bacilysin, bacillibactin and subtilosin A BGC while a fraction (8 out of 21 strains) harbors a mojavensin A BGC (Figure 6). Mojavensin A production has been confirmed in *B. halotolerans* strains Cal.l.30, FJAT-2398, NRLL B-41618T, NRLL B-41617T and ATCC 25096T [48]. All the *B. halotolerans* strains harboring or lacking the mojavensin A BGC cluster on neighboring but distinct branches of the phylogenetic tree (Figure 6). The inter-genomic relatedness among the *B. halotolerans* strains (ANI and dDDH values) showed significant variation. All *B. halotolerans* strains harboring the mojavensin A BGC exhibit a relatively very high value of dDDH (92–93%) and ANI (98–99%) among them, while exhibit a relatively lower dDDH (80-82%) value with all the bacterial strains (e.g. *B. halotolerans* F43-1, *B. halotolerans* KKD1) lacking the mojavensin A BGC (Figure 4).

### 3.6. Analysis of CAZyme Genes in B. halotolerans Stain Cal.l.30 Genome

Genome mining revealed the presence of 119 CAZymes encoding enzymes that target key components of the plant and fungal cell wall mainly cellulose, chitin and hemicellulose. There were 54 glycoside hydrolases (GHs), 38 glycosyltransferases (GTs), 7 polysaccharide lyases (PLs), 14 carbohydrate esterases (CEs), and 1 auxiliary activity (AAs), as well as 13 carbohydrate-binding modules (CBMs) (Table 2). A large fraction (25.2%) of the CAZyme proteins possess amino-terminal signal peptides to mediate protein export through the cytoplasmic membrane, indicating that these are secreted enzymes. The genome of Cal.l.30 is characterized by an abundance of genes encoding for possible antifungal CAZymes (secreted and non-secreted) in the GH families, such as chitinase (GH18), chitosanase (GH46), glucanase (GH16, GH51, GH64) and β-glucosidase (GH1, GH3), which have the potential to inhibit the growth of plant fungal pathogens (Table 2).

### 3.7. Constitutive Production and Secretion of Agar-Diffusible Antifungal Metabolites of Cal.l.30 When Grown on a Solid Surface

**Figure 7:**
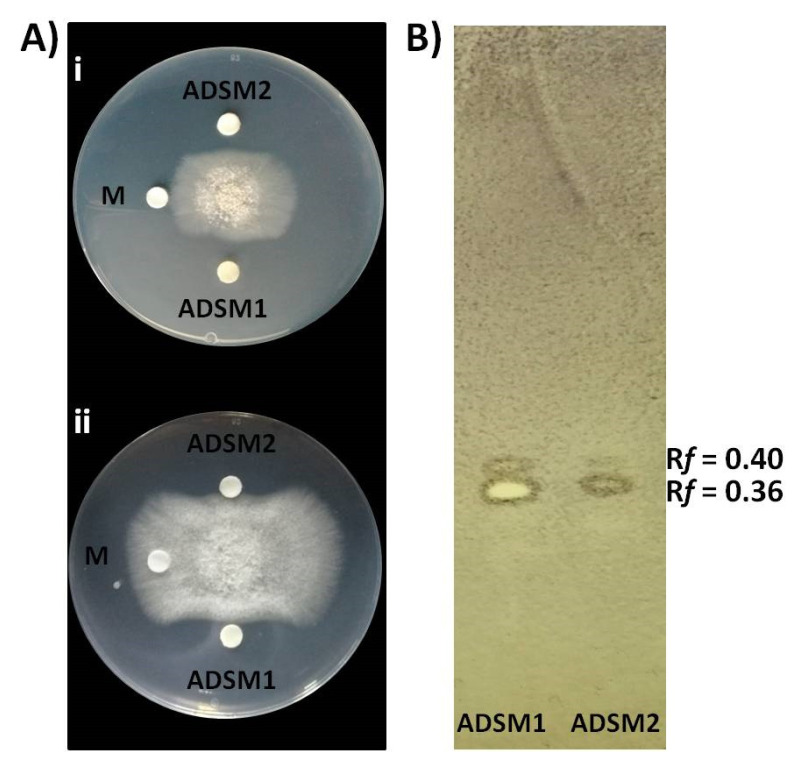
Inhibitory effect of agar-diffusible secondary metabolites (ADSM) of Cal.l.30 against *B. cinerea*. (**A**) Confrontation well-diffusion assay at 3 d (**i**) and 6 d (**ii**) of interaction. ADSM1 and ADSM2 represent agar-diffusible secondary metabolites of Cal.l.30 when grown singly and together with *B. cinerea*, respectively, while M is the solvent of methanol used as negative control. (**B**) TLC-bioautography using ADSM1 and ADSM2 of Cal.l.30.

Cal.l.30 ethyl acetate extracts of agar-diffusible secreted metabolites produced when Cal.l.30 is grown singly (ADSM1) or during confrontation with *B. cinerea* (ADSM2), were evaluated for their antifungal activity using agar-plate diffusion base assay and TLC-bioautography assay. Both ADSM1 and ADSM2 could effectively constrain the growth and expansion of fungal mycelium (Figure 7A). Furthermore, TLC-bioautography, using *B. cinerea* as an indicator, revealed that both ADSM1 and ADSM2 form a strong inhibition spot (suppressed conidial germination) with identical R*f* values (0.36 and 0.40), showing that Cal.l.30 biosynthesizes and secretes bioactive metabolites when grown individually on a solid surface (Figure 7B).

Ultra-high-performance liquid chromatography coupled to Q Exactive Orbitrap High-Resolution Mass Spectrometry (UHPLC-HRMS) was employed for the putative annotation of secondary (antifungal) metabolites biosynthesized and secreted by strain Cal.l.30 when grown singly on a solid surface. The UHPLC-HRMS chemical analysis revealed the presence of different types of secondary metabolites with antimicrobial/ antifungal activity, including the lipopeptides, mojavensin A, fengycin and surfactin, the polyene diamide polyketide bacillaene, the intermediate metabolite of bacilysin biosynthetic pathway, L-dihydroanticapsin and the siderophore bacillibactin (Table 3).

**Figure 8:**
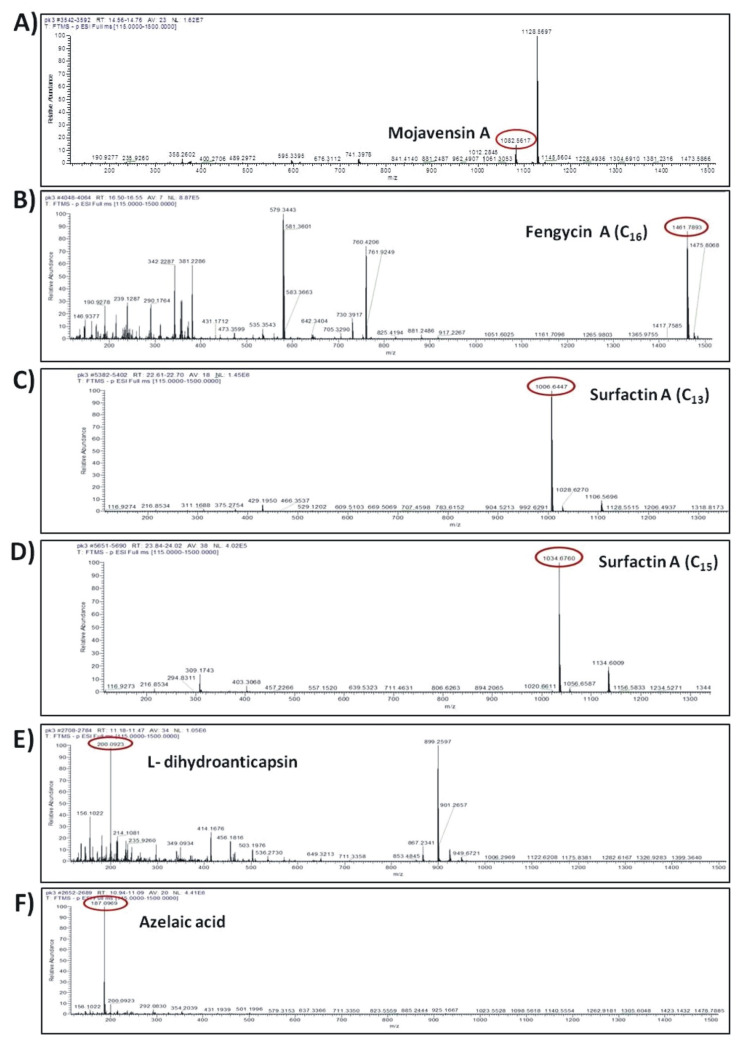

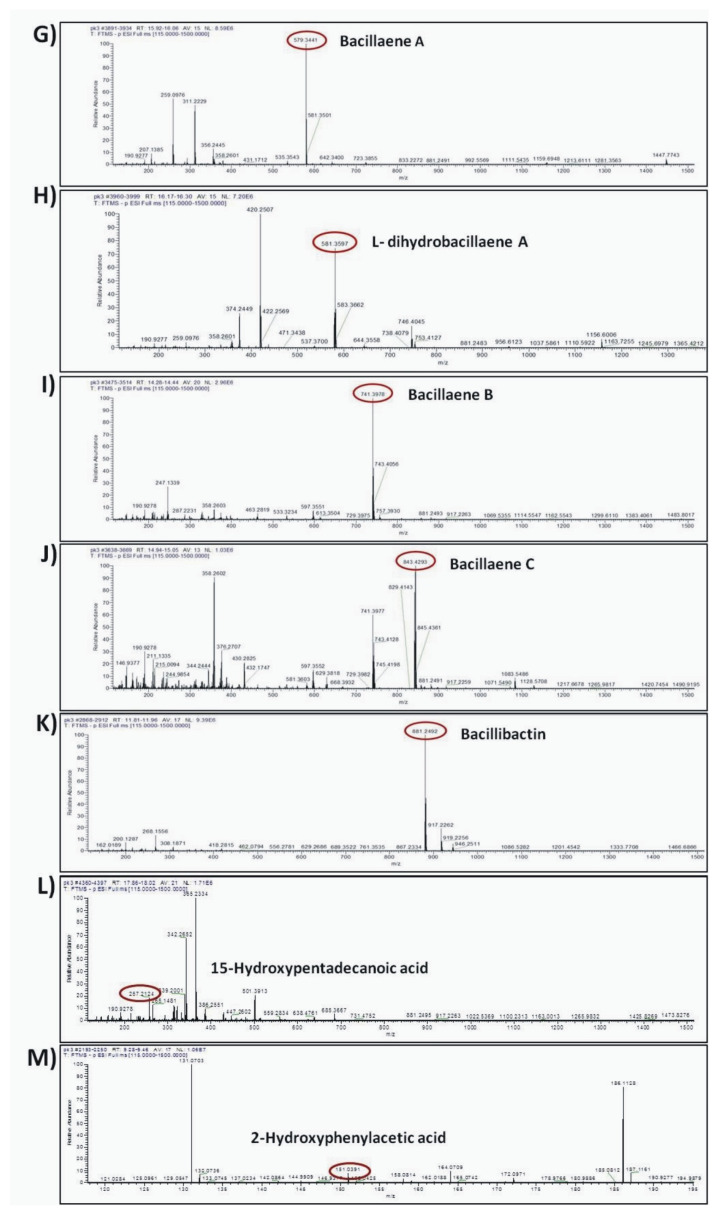
Agar-diffusible secondary metabolites secreted by *B. halotolerans* Cal.l.30 and annotated through UHPLC-HRMS analysis. The compounds were putatively annotated based on isotope distribution and the accurate mass (±5 ppm). The precise masses revealed the presence of compounds (**A**) mojavensin A, (**B**) fengycin A, (**C**) Surfactin A C_13,_ (**D**) Surfactin A C_15_, (**E**) L-dihydroanticapsin (**K**) bacillibactin, (**G**–**J**) four analogues of bacillaene, known as bacillaene A, dihydrobacillaene, bacillaene B and bacillaene C, respectively, (**F**,**L**,**M**) azelaic acid, 15-hydroxypentadecanoid acid and 2-hydroxyphenylacetic acid, respectively.

The compounds were putatively annotated based on isotope distribution and the accurate mass (±5 ppm). The precise masses obtained from ions *m/z* 1082.5617 [M–H]- and *m/z* 1461.7893 [M–H]- revealed the presence of two compounds with molecular formulas C_50_H_77_N_13_O_14_ and C_72_H_110_N_12_O_20_ annotated as mojavensin A and fengycin A, respectively (Figure 8A,B). In addition, HRMS analysis revealed the presence of ions with *m/z* 1006.6447 [M–H]- and *m/z* 1034.6760 [M–H]- corresponding to surfactin A analogues with molecular formula of C_51_H_89_N_7_O_13_ (Surfactin A C_13_) and C_53_H_93_N_7_O_13_ (Surfactin A C_15_), respectively (Figure 8C,D). The compounds with *m/z* 881.2492 [M–H]- and *m/z* 200.0923 [M–H]- were assigned to the siderophore bacillibactin and L-dihydroanticapsin, respectively (Figure 8E,K). The compounds with *m/z* 579.3441 [M–H]-, *m/z* 581.3597 [M–H]-, *m/z* 741.3978 [M–H]- and *m/z* 843.4293 [M–H]- were assigned to the four analogues of bacillaene, known as bacillaene A, dihydrobacillaene, bacillaene B and bacillaene C, with molecular formulas C_34_H_48_N_2_O_6_, C_34_H_50_N_2_O_6_, C_40_H_58_N_2_O_11_ and C_44_H_64_N_2_O_14_, respectively (Figure 8G–J). 

In addition, other compounds, such as azelaic acid, and the fatty acids 15-hydroxypentadecanoid acid and 2-hydroxyphenylacetic acid, with known antioxidant, antibiotic and/or antifungal activity were also detected in the ADSM1 fraction (Table 3). Ion signals with *m/z* 187.0969 [M–H]-, *m/z* 257.2124 [M–H]- and *m/z* 151.0391 [M–H]- were assigned to azelaic acid, 15-hydroxypentadecanoid acid and 2-hydroxyphenylacetic acid with molecular formulas C_9_H_6_O_4_, C_15_H_30_O_3_ and C_8_H_8_O_3_, respectively (Figure 8F,L,M).

## 4. Discussion

The ecological challenges posed by the extensive use of chemical fungicides to control plant pathogens have reinforced the need for replacing them with biopesticides against plant pathogens [60]. The application of microorganisms and especially bacteria in agriculture has significantly increased in recent years, making it necessary to find strong and effective BCAs. Endophytic bacteria derived from medicinal plants are an important source of potential BCAs, due to the diverse competing mechanisms they have developed in order to adapt and survive endophytically [44,61]. *C. officinalis* is one of the most important medicinal plants, used in traditional medicine since 12 BC, due to its multiple antimicrobial properties [62]. As far as we know, only one study has been conducted on the endophytic bacterial community of the medicinal plant *C. officinalis*, from which a very small number of bacteria (five strains) were isolated and only from the leaves and shoots of the plant [63].

As presented in a previous study of ours, a total number of 119 cultivable endophytic bacterial strains were isolated from different plant parts such as roots, leaves and flowers of the medicinal plant *Calendula officinalis*, from which twenty-four morphologically distinct endophytic bacterial strains of the genus *Bacillus* were identified based on 16S rRNA sequencing analysis [25]. After co-cultivation with *Arabidopsis thaliana* seedlings, the majority of *Bacillus* strains positively affected plants’ morphological characteristics such as total fresh weight, primary root length, lateral root number and root hair number [25]. In the current study, the most promising *Bacillus* isolates, *B. halotolerans* strains Cal.l.30, Cal.f.4, Cal.l.11, Cal.f.2.1, Cal.r.11, *B. velezensis* strain Cal.r.29 and *B. subtilis* strain Cal.r.19, were further selected and studied for their possible antifungal activity. Among them, *Bacillus velezensis* Cal.r.29, *B. halotolerans* Cal.l.30 and *B. halotolerans* Cal.r.11 prevented *B. cinerea* more efficiently by creating the strongest inhibition zone in between, under in vitro dual culture assay. Bacteria of the genus *Bacillus* stand out for their antagonistic activity due to their multiple antimicrobial secondary metabolites and modes of action, with secreted lipopeptides being the most crucial [2,8]. Previous studies have shown that the inhibition zone created during the microbial interaction consists of lytic enzymes and accumulated secreted antimicrobial compounds with biosurfactants, mainly lipopeptides, being mostly responsible for phytopathogen suppression [52]. The secretion of biosurfactants takes place not only on solid and semi-solid substrates, but also on liquid growth medium, maintaining their strong antimicrobial activity [52]. Biosurfactants are molecules that play an important role in antagonistic attributes of microbial BCAs because they facilitate colonization, they may have direct antimicrobial properties or elicit an immune response in plant tissues that makes the host less susceptible to subsequent infection [10]. In the present study, all seven *Bacillus* strains that created a strong inhibition zone against *B. cinerea*, were also positive for secreting biosurfactants into their liquid culture with their supernatant showing excellent drop collapse ability.

Although endophytic bacteria of the *Bacillus* species have emerged as an alternative promising strategy to control plant pathogens [8], the discovery and scrutiny of novel endophytic *Bacillus* spp. can provide new options in the field of food and crop protection, specifically for the development of efficient microbial biocontrol agents to suppress post and preharvest fungal plant diseases [10,20]. In the current study, *B. halotolerans* was the predominant species among the *Bacillus* species isolated from *C. officinalis*, with strain Cal.l.30 being the most promising BCA candidate. *B. halotolerans* is a newly characterized species within the *Bacillus* genus [64]. Thus far, a limited number of plant-associated *B. halotolerans* strains have been isolated, and even fewer have been evaluated for their potential to act as BCAs against plant pathogens [11,12,13,18]. 

In the present study, an integrative approach coupling genome mining and metabolic profiling has been applied to decipher the potential of the novel endophyte *B. halotolerans* Cal.l.30 as a BCA against the postharvest pathogen *B. cinerea*. Genome mining revealed that a considerable part (13%) of the Cal.l.30 genome is predicted to be devoted to the biosynthesis of secondary metabolites compared to the approximately 9% identified in the other *B. halotolerans* strains [11,12]. *B. halotolerans* Cal.l.30, in addition to the fengycin/plipastatin, surfactin, bacillaene, bacilysin, bacillibactin and subtilosin A BGCs found in all *B. halotolerans* strains, contains also both the mojavensin A and FAS-PKS gene clusters. Phylogenomic analysis revealed that all the *B. halotolerans* strains harboring or lacking the mojavensin A BGC cluster in well-separated distinct branches of the phylogenetic tree. Furthermore, *B. halotolerans* strains harboring the mojavensin A BGC share above 91% dDDH similarities with each other but only 80–82% with other *B. halotolerans* strains, a value very close to the boundary of 79–80% dDDH for subspecies delineation [65]. Thus, it could be argued that the acquisition of gene clusters may lead to ecological diversification and speciation [66,67]. Whether this speciation process may be related to endophytic behavior is not yet known [68]. 

The mojavensin A and FAS-PKS BGCs’ diversity across *B. halotolerans* strains suggests that both BGCs may have been acquired via recombination and horizontal gene transfer processes [69,70,71]. Our in silico analysis revealed that *B. halotolerans* strains contain ‘hot spots’ at specific location of their genome, allowing the preferential recombination at non-homologous regions that are flanked by homologous regions with the net result being the incorporation of mojavensin A BGC during a horizontal gene transfer (HGT) event [72]. The mechanism only requires housekeeping of recombination functions and exogenous DNA with homology to the flanking core genes [72,73]. The evolutionary origin of these novel BGCs is not known; however, the presence of almost identical mojavensin A BGC in both *B. halotolerans* Cal.l.30 and the *B. cabrialesii* genome, and the identical FAS-PKS BGC in *B. halotolerans* Cal.l.30 and *B. velezensis* SQR9, clearly indicates that these clusters may have originated via an HGT event from a donor closely related to the *B. subtilis* species. 

Horizontal BGC transfer usually results in the production of an existing compound in a new, typically distantly related organism [74]. Thus, the acquisition of BGCs by the Cal.l.30, as well as the potential endowment with new traits, also provides the opportunity to improve upon an existing trait, generating synergistic effects between the corresponding secondary metabolites. As a result, strain Cal.l.30 may compete with other members of the population, and the associated genome can theoretically sweep through the population [75].

The ecological role of mojavensin A BGC and the FAS-PKS BGC products in Cal.l.30 is not yet known. A few studies have aimed to explore the ecological role of the biosynthetic products of mojavensin A and FAS-PKS BGCs [51,76]. Purified mojavensin A from *B. mojavensis* B0621A displayed moderate antagonism and dose-dependent activity against *F. oxysporum* [76], while mojavensin A produced by the actinomycete *Gordonia strain* WA 4-31 showed adequate antimicrobial activity against both *Aspergillus nidulans* and *Escherichia coli* [77]. Interestingly, mojavensin A and a cyclic (leucine–leucine) dipeptide had a synergistic effect and showed enhanced antimicrobial activity [77]. On the other hand, the novel cluster (a FAS-PKS biosynthetic gene cluster) found in SQR9 appeared to be involved in the biosynthesis of the novel long-chain fatty acid(s) (C_15_H_30_O_3_ and/or C_17_H_34_O_3_) such as bacillunoic acid(s), presenting an adequate antifungal and antibacterial activity [51]. Furthermore, inactivation of the FAS-PKS BGC appeared to reduce root colonization ability of *B. velezensis* SQR9 over closely related rhizobacteria [51]. Following the aforementioned observation, similar or identical compounds may be produced by Cal.l.30 since 15-hydroxypentadecanoid acid with the chemical formula C_15_H_30_O_3_ was identified.

In comparison to other *B. halotolerans* strains, Cal.l.30 is a novel and powerful strain due to its ability to biosynthesize and secrete a significant number of secondary metabolites and strong antimicrobial compounds. Among the compounds identified after UHPLC-ESI HRMS analysis are C_16_ fengycin A, surfactin A (C_13_ surfactin A and C_15_ surfactin A), mojavensin A, four analogues of the antibiotic polyketide bacillaene (bacillaene A1, bacillaene B1, bacillaene C2 and dihydrobacillaene A), the iron chelating compound bacillibactin, the active substance azelaic acid, and the fatty acids 15-hydroxypentadecanoid acid and 2-hydroxyphenylacetic acid. In addition, the secondary metabolite L-dihydroanticapsin was identified in *B. halotolerans* Cal.l.30, which is the precursor of the potent antibiotic bacilycin [55]. Bacilycin is an important dipeptide with strong antibacterial and antifungal activity [51]. 

One of the most important observations in our study was the ability of Cal.l.30 to secrete the iturinic-type lipopeptide mojavensin A. Metabolic analysis of Cal.l.30, confirmed the results of genomic analysis corresponding to the lipopeptide of mojavensin A (C_50_H_77_N_13_O_14_), with an *m/z* value of 1082.5617. This result is in agreement with Ma and colleagues who first isolated and identified mojavensin A in *B. mojavensis* B0621A [49].

Of particular interest was the detection of the polyketide bacillaene, biosynthesized by the endophyte Cal.l.30. Although it is a chemically unstable secondary metabolite, sensitive to light, oxygen and even room temperature [54], we were able to detect four analogues of the compound, identified as bacillaene A, dihydrobacillaene A, bacillaene B and bacillaene C [54]. Due to the sensitivity of the polyketide, a limited number of literature reports have been able to highlight the antibacterial activity of the compound and its analogues, as well as their inhibitory effect against fungi in vitro [78,79,80,81]. Furthermore, bacillaene can accelerate self-biofilm development of *B. subtilis* at sub-inhibitory concentrations [54], inhibit biofilm formation and disperse pre-established biofilm by other bacteria [82], protect *B. subtilis* from predation by other bacteria [83] and may modulate the production of secondary metabolites by competing bacteria [84].

One of the most significant compounds secreted and identified in *B. halotolerans* Cal.l.30 is the active substance azelaic acid (AzA). Thus far, only a few reports have been found on the biosynthesis of AzA by bacteria, and only two by *Bacillus* species and, specifically, *B. velezensis* Bvel1 and *B. halotolerans* Hil4 [18,26,56]. AzA is a nine-carbon dicarboxylic acid, which has been reported primarily in plants as a mobile signaling molecule that activates plant defense mechanisms [85]. Specifically, the produced azelaic acid triggers the accumulation of SA in plants by activating SAR in order to treat phytopathogenic microorganisms, either in the contact tissue or systemically [86,87]. 

The current study establishes the antagonistic endophytic bacterial strain Cal.l.30 as a strong inhibitor of the necrotrophic phytopathogenic fungus *B. cinerea*, under both in vitro (dual culture assay) and ex vivo conditions (detached fruit assay). The application of bacteria on plant tissues is an important method of selecting competing BCAs, able to protect plants from pathogenic microorganisms. Strain Cal.l.30 was able to suppress *B. cinerea* and significantly reduce disease incidence and disease severity index of gray mold when applied on tomato cherry fruits and grape berries, in comparison to control treatments. Moreover, the application of Cal.l.30 cell free supernatant on both the fruit tissues significantly inhibited the spread of the phytopathogen, confirming the existence of bioactive secondary metabolites in its liquid culture.

A necessary step for an efficient BCA inoculation and biological control is the successful colonization of the BCA candidate at the point of formulation and its effective competition for nutrients and space against the host microbial community [24,88,89]. In the present study, strain Cal.l.30 successfully colonized both of the plant tissues tested, keeping a high cell density until the last day of observation. It is known that the cyclic lipopeptide surfactin, produced by bacteria of the genus *Bacillus*, is directly involved in the movement, biofilm formation and colonization of bacteria on surfaces and is less referred to as a strong antimicrobial compound [90,91,92]. Among surfactin’s compounds, isoform C15 surfactin A, which was also identified in strain Cal.l.30, is a particularly potent antibiotic lipopeptide against a large number of phytopathogenic fungi, including *B. cinerea* [93,94,95]. In contrast to the surfactin lipopeptide, which is mainly involved in biofilm formation and bacterial colonization, the cyclic lipopeptides of the iturin and fengycin family are the major antimicrobial compounds of the *Bacillus* species [49,50,91,92,96]. Both genomic and metabolomic analysis of Cal.l.30 indicated the production of surfactin (C13 surfactin A and C15 surfactin A), fengycin (C16 fengycin A) and mojavensin A biosurfactants, enhancing their role in the colonization of Cal.l.30 on tomato fruits and grape berries and antagonistic activity. It is worth mentioning that bacillaene compounds, in addition to their antimicrobial activity, accelerate the production of biofilm for a better bacterial cell self-protection [54]. Therefore, the production of bacillaene may have played a crucial self-protective role for strain Cal.l.30, increased biofilm formation for a successful ex vivo colonization and combated potential competing microorganisms.

As a result of successful colonization, Cal.l.30 likely reduced *B. cinerea* access to essential nutrients, such as iron. In silico analysis of strain Cal.l.30 highlighted the genes involved for the biosynthesis of the siderophore bacillibactin, while metabolomics enhanced the above result by identifying the compound (C_39_H_42_N_6_O_18_). Iron is one of the key ingredients in the formation of the bacterial biofilm as it stabilizes the matrix of polysaccharides involved in its formation [97]. Therefore, the binding of available iron significantly increases biofilm formation and, consequently, the colonization of bacteria at the site of inoculation. Absorption of available iron is carried out by the production of bacterial iron chelating compounds, which significantly reduce the available iron to phytopathogenic fungi, an element necessary for their spore germination, growth and, ultimately, their infectivity [98,99,100]. Thus, the production of bacterial iron chelating compounds in comparison to the total number of antimicrobial compounds, enhances their competitiveness [101].

A significant number of scientific reports have focused on the emergence of bacterial lipopeptides (LPs), polyketides (PKs), lytic enzymes, phytohormones, volatiles, iron compounds and various proteins (e.g. flagellin, flg22) as strong ISR elicitors [10,102,103,104]. Waewthongrak and colleagues presented ISR induction of mandarin fruits after the application of both the endospores and bioactive supernatant of *B. subtilis* ABS-S14, resulting in an efficient protection against the pathogenic fungus *Penicillium digitatum* [105]. Fragments of chitosan, as a result of chitinolytic enzymes action, are known to elicit plant defense reactions both locally and systemically, including the stimulation of the production of small molecules such as jasmonic acid and abscisic acid [106]. Additionally, lipopeptides, such as plipastatin, bacillomycin L and surfactin produced by antagonistic *Bacillus* sp., have also been reported as elicitors for inducing disease resistance within plant hosts [107,108]. Phenylacetic acid produced and secreted by *B. fortis* IAGS162 may act as an ISR elicitor contributing to tomato plants’ resistance against *Fusarium* wilt disease [109]. Furthermore, a recent study demonstrated that medium-chain 3-hydroxy fatty acids ((R)-3-hydroxydecanoic acid) trigger immunity in *Arabidopsis* [110].

## 5. Conclusions

This study introduced the novel *B. halotolerans* Cal.l.30 strain, which is endowed with a rich arsenal of secondary metabolite biosynthetic gene clusters, equipped with a large repertoire of CAZyme genes involved in antifungal activity, and armed with the capacity to produce and secrete secondary metabolites and other novel compounds that are involved in antibiosis and/or host plant systemic resistance induction and the successful colonization of host plant tissues. In many cases, fungal pathogens such as *B. cinerea* and *Colletotrichum acutatum* infest fruits (at the flower stage) in the field, and these latent infections may become active at pre and/or postharvest stage [111]. Based on in vitro and ex vivo assays, *B. halotolerans* Cal.l.30 effectively controlled the ingression and expansion of the postharvest fungal pathogen *B. cinerea* in both tomato fruits and grape berries. In this context, it will be of interest to explore the prospects of the endophytic strain *B. halotolerans* Cal.l.30 as a biological control agent of pre and postharvest fungal diseases. 

## Figures and Tables

**Table 1:** Biosynthetic gene clusters (BGCs) involved in biosynthesis of secondary metabolites of bacterial strain *B. halotolerans* Cal.l.30, detected by antiSMASH server.

Cluster	BGC Type	Compound	MIBiG ID (Similarity %)	Predicted Size (bp)
1	NRPS	Surfactin	BGC0000433_c1 (86%)	65.394
2	T1PKS-NRPS	Kalimantacin A	BGC0001532_c1 (20%)	117.825
3	Terpene	Unknown	-	20.426
4	TransAT-PKS-NRPS	Bacillaene	BGC0001089_c1 (100%)	114.235
5	TransAT-PKS-NRPS	Fengycin/ Mojavensin A	BGC0001095_c1 (100%)	129.752
6	Terpene	Unknown	-	21.898
7	T3PKS	Unknown	-	41.097
8	NRPS	Bacillibactin	BGC0000309_c1 (100%)	49.738
9	Other	Bacilysin	BGC0001184_c1 (100%)	41.418
10	Sactipeptide	Subtilosin A	BGC0001184_c1 (100%)	21.612

**Table 2:** Distribution of CAZymes gene families in the genome of *B. halotolerans* Cal.l.30.

CAZyme Categories	Family	Activity	Gene Copy Numbers
Glycoside Hydrolases (GH)	GH0	-	1
	GH1	β-glucosidase	4
	GH3	β-glucosidase	1
	GH4	maltose-6-phosphate glucosidase	4
	GH5	cellulase	1
	GH11	endo-β-1,4-xylanase	1
	GH13	α-amylase	9
	GH16	xyloglucan:xyloglucosyltransferase	1
	GH18	chitinase	4
	GH23	lysozyme type G	4
	GH26	β-mannanase	1
	GH30	endo-β-1,4-xylanase	1
	GH32	invertase	3
	GH42	β-galactosidase	2
	GH43	β-xylosidase	4
	GH51	endoglucanase	2
	GH53	endo-β-1,4-galactanase	1
	GH46	chitosanase	2
	GH65	α,α-trehalase	1
	GH68	levansucrase	1
	GH73	lysozyme	2
	GH105	unsaturated rhamnogalacturonyl hydrolase	2
	GH126	α-amylase	1
	GH171	peptidoglycan β-N-acetylmuramidase	1
Glycosyl Transferases (GT)	GT0	-	4
	GT1	UDP-glucuronosyltransferase	3
	GT2	cellulose synthase	13
	GT4	sucrose synthase	7
	GT5	glycogen glucosyltransferase	1
	GT8	α-1,3-galactosyltransferase	1
	GT26	β-N-acetyl mannosaminuronyltransferase	1
	GT28	1,2-diacylglycerol 3-β-galactosyltransferase	3
	GT35	glycogen	1
	GT51	murein polymerase	4
Carbohydrate Esterases (CE)	CE4	acetyl xylan esterase	7
	CE7	acetyl xylan esterase	1
	CE9	N-acetylglucosamine 6-phosphate deacetylase	2
	CE12	pectin acetylesterase	3
	CE14	N-acetyl-1-D-myo-inosityl-2-amino-2-deoxy-α-D-glucopyranoside deacetylase	1
Polysaccharide Lyases (PL)	PL1	pectate lyase	2
	PL3	pectate lyase	1
	PL9	pectate lyase	1
	PL11	rhamnogalacturonan endolyase	2
	PL26	rhamnogalacturonan exolyase	1
Auxiliary Activities (AA)	AA10	lytic polysaccharide monooxygenases	1
Carbohydrate-Binding Modules (CBM)	CBM16	binding to cellulose and glucomannan	1
	CBM50	attached to various enzymes from families GH18, GH19, GH23, GH24, GH25 and GH73	11
	CBM63	bind cellulose	1

**Table 3:** Secondary metabolites biosynthesized by *B. halotolerans* Cal.l.30 and putatively annotated through UHPLC-HRMS analysis.

Antibiotic Compounds	Molecular Formula	Experimental *m/z*	RT (min)	Δppm	Adduct	Reference or Source
Mojavensin A	C_50_H_77_N_13_O_14_	1082.5617	14.66	-1.13	[M–H]-	[49]
Surfactin A C_13_	C_51_H_89_N_7_O_13_	1006.6447	22.699	1.23	[M–H]-	[50]
Surfactin A C_15_	C_53_H_93_N_7_O_13_	1035.683	23.945	0.57	[M–H]-	[51]
Fengycin A C_16_	C_72_H_110_N_12_O_20_	1461.7893	16.51	1.19	[M–H]-	[52]
Bacillaene A	C_34_H_48_N_2_O_6_	579.3441	16.365	2.13	[M–H]-	[53]
dihydrobacillaene A	C_34_H_50_N_2_O_6_	581.3597	16.24	2.04	[M–H]-	[53]
Bacillaene B	C_40_H_58_N_2_O_11_	741.3978	14.357	2.85	[M–H]-	[54]
Bacillaene C	C_44_H_64_N_2_O_14_	843.4293	14.984	2.27	[M–H]-	[54]
L-dihydroanticapsin	C_9_H_15_NO_4_	200.0923	11.395	2.83	[M–H]-	[55]
Azelaic acid	C_9_H_16_O_4_	187.0969	11.022	2.21	[M–H]-	[56]
Bacillibactin	C_39_H_42_N_6_O_18_	881.2497	11.88	2.85	[M–H]-	[57]
15-hydroxypentadecanoid acid	C_15_H_30_O_3_	257.2124	17.953	4.97	[M–H]-	[58]
2-hydroxyphenylacetic acid	C_8_H_8_O_3_	151.0391	9.381	0.86	[M–H]-	[59]

## Data Availability

The *Bacillus halotolerans* strains Cal.l.30, Cal.f.4 and Cal.l.11 whole genome projects are available in the NCBI database under the accession numbers JAEACK000000000, JAEACI000000000 and JAEACJ000000000(GenBank), respectively, and SAMN16949411, SAMN16949417, SAMN16949418 (BioSample) and PRJNA681331, PRJNA681333, PRJNA681332 (BioProject), respectively.
